# Giant hemorrhagic and abscessed pediatric high-grade glioma with osteolysis and transcalvarial herniation: A unique presentation and literature review

**DOI:** 10.1016/j.ijscr.2025.111748

**Published:** 2025-08-07

**Authors:** Mehdi Borni, Marouen Taallah, Brahim Kammoun, Hatem Daoud, Houda Belmabrouk, Mohamed Zaher Boudawara

**Affiliations:** a Department of Neurosurgery, Habib Bourguiba University Hospital, Sfax, Tunisia; b Department of Neurosurgery, Mohamed Ben Sassi University Hospital, Gabes, Tunisia

**Keywords:** Pediatric high grade glioma, Abscess, Transcalvarial herniation, Surgery, Chemotherapy

## Abstract

**Introduction:**

High-grade gliomas (HGGs) in very young children are rare, with intratumoral abscess formation being exceptionally uncommon and primarily reported in adults. We present a unique pediatric case with this rare complication.

**Case report:**

A 5-month-old female infant, born after a complicated pregnancy, presented with rapidly progressive intracranial pressure. Imaging revealed a large, hemorrhagic, left parieto-occipital lesion with osteolysis and subsequent transcalvarial herniation. Intraoperatively, a culture-negative abscess was found within the tumor. Initial biopsy suggested a low-grade glioma, but final histopathology confirmed a high-grade glioma.

**Discussion:**

The combination of a giant, abscessed, and osteolytic HGG leading to transcalvarial herniation in a young infant is unprecedented. Intratumoral abscesses in pediatric HGGs are not previously documented, making this case a significant addition to the literature. The sterile abscess may be linked to tumor necrosis or the patient's perinatal history. This atypical presentation underscores the diverse nature of pediatric HGGs.

**Conclusion:**

This first reported case of a giant, hemorrhagic, osteolytic, and abscessed pediatric HGG with transcalvarial herniation highlights an exceptionally rare and aggressive presentation in a very young infant. It emphasizes the need for considering unusual features in pediatric brain tumors and warrants further investigation into the pathogenesis and management of such complex lesions.

## Highlights

• First Pediatric Abscessed HGG: Reports the first documented case of a giant, osteolytic high-grade glioma (HGG) with transcalvarial herniation and an intratumoral abscess in a 5-month-old infant.
• Atypical Infantile Presentation: Highlights an unusually aggressive HGG presentation in infancy, characterized by rapid progression and significant tumor burden.
• Diagnostic Challenges: Underscores the difficulties in HGG diagnosis and management in infants, particularly with initial misdiagnosis in resource-limited settings.
• Sterile Intratumoral Abscess: Documents a rare culture-negative intratumoral abscess, suggesting a sterile process linked to tumor necrosis/hemorrhage.
• Unique Pediatric Insights: Offers valuable insights into rare and aggressive HGG manifestations in the youngest patients, emphasizing the need for high clinical suspicion.

## 1 Introduction

Glial tumors represent over 50 % of solid central nervous system (CNS) tumors in the pediatric population [1]. While less common than their low-grade counterparts, pediatric high-grade gliomas (HGGs) have an estimated incidence of 1.1–1.78 per 100,000 [2,3] and are the leading cause of tumor-related mortality in this age group [1,2].

Although pediatric HGGs share morphological features with adult forms [4], they possess distinct molecular characteristics and differ in anatomical distribution. Pediatric HGGs more frequently occur in midline locations, unlike the predominantly hemispheric involvement seen in adults [3]. The 2021 World Health Organization (WHO) classification of CNS tumors increasingly incorporates molecular markers due to their critical role in diagnosis, prognostication, and understanding tumor biology [5,6]. Clinically, childhood HGG presentations are primarily determined by tumor location and patient age, often manifesting as increased intracranial pressure, neurological deficits, and focal neurological symptoms [7,8].

To our knowledge, intratumoral abscess formation in pediatric HGGs has not been previously reported; existing cases are documented exclusively in adult populations [9]. This report describes the first documented instance of a giant, hemorrhagic, abscessed, and osteolytic high-grade glioma in a 5-month-old female infant, resulting in transcalvarial herniation. The infant was born via Cesarean section for fetal distress secondary to intra-amniotic infection after an unmonitored pregnancy. This unique presentation highlights the potentially complex and underrecognized factors in such cases.

The work has been reported in line with the SCARE criteria [10].

## 2 Case report

A 5-month-old female infant, the first child of non-consanguineous parents under 20 years of age, was admitted to our neurosurgery department with rapidly progressive signs of raised intracranial pressure (ICP). Her symptoms included macrocrania, morning non-bilious vomiting, and increasing lethargy. The infant was born at normal weight via Cesarean section due to fetal distress, marked by intra-amniotic infection and meconium-stained amniotic fluid. Her mother reported increasing irritability and inconsolable, high-pitched crying in the days leading up to admission. The pregnancy was unmonitored, and the infant had an incomplete vaccination status with no regular postnatal consultations. At birth, her Apgar scores were 9 at 1 min and 10 at 5 min, with a head circumference of 35 cm (13.77 in.).

Upon initial examination, the infant presented with significant hypotonia, drowsiness, and a setting sun sign. Her head circumference had markedly increased to 40.5 cm (+3 Standard Deviations), and she had a prominent, bulging anterior fontanelle. No focal neurological deficits, strabismus, or pupillary abnormalities were observed.

**Fig. 1:**
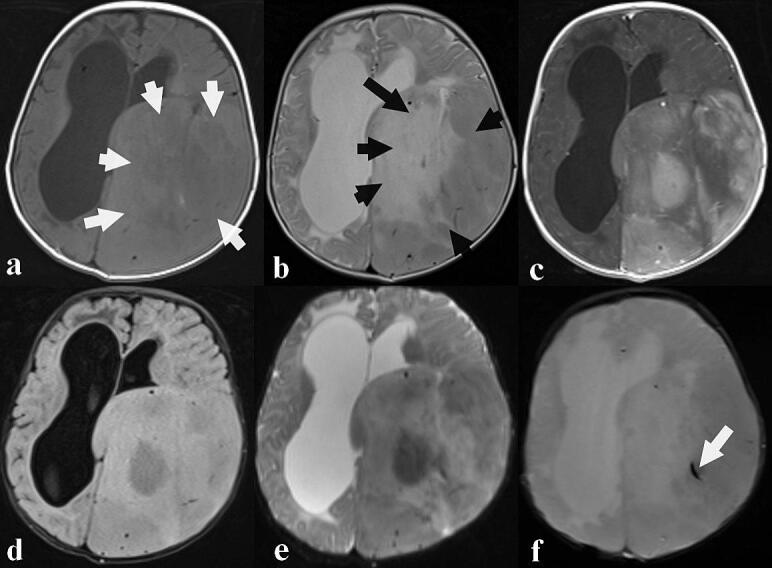
Axial brain MRI showing a large left parieto-occipital space-occupying lesion with mixed signal on T1-weighted sequence (some areas in hyposignal and others in isosignal) (**a**, white arrows) and of heterogeneous signal on T2-weighted sequence (**b**, black arrows) and with heterogeneous enhancement after gadolinium chelate injection (**c**). Note a slight perilesional edema all around on the T2 fluid attenuated inversion recovery sequence (**d**). The tumor was compressing the midbrain, cerebral aqueduct and the third ventricle and which is responsible for a significant right lateral ventricle dilatation and moderate frontal horn of the left lateral ventricle dilatation. There was no hypersignal on diffusion image (**e**) and the T2 sequence in gradient echo showed some stigmata of bleeding (**f**, white arrow).

Complete blood count, electrolytes, and hemostatic screening tests were all unremarkable, as was a chest X-ray. A brain MRI (Fig. 1) revealed a large (11.8 × 8.5 × 9.7 cm), left parieto-occipital space-occupying lesion. The tumor appeared to originate intraventricularly at the junction of the ventricular, temporal, and occipital horns of the left lateral ventricle, invading the adjacent parietal and occipital brain parenchyma, suggestive of choroid plexus development. It showed mixed T1 and heterogeneous T2 signal intensity, with heterogeneous enhancement post‑gadolinium, indicating significant tumor necrosis. Mild perilesional edema was present. The mass effect compressed the midbrain, cerebral aqueduct, and third ventricle, causing significant dilatation of the right lateral ventricle and moderate dilatation of the left lateral ventricle's frontal horn. Hyposignal on T2-weighted gradient echo sequences suggested areas of calcification and/or hemorrhage.

**Fig. 2:**
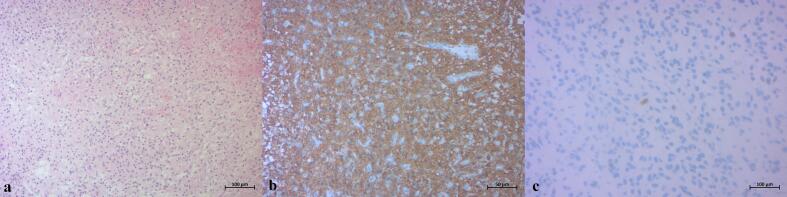
Photomicrograph of the initial biopsy specimen (Hematoxylin and Eosin staining, 100× original magnification) (Fig. 2a) revealed a low to moderate density proliferation of glial cells. These cells exhibited relatively uniform nuclei with minimal to mild cytonuclear atypia, and notably, no areas of necrosis or significant mitotic activity were identified in this sample. Immunohistochemical analysis demonstrated strong and diffuse cytoplasmic expression of Glial Fibrillary Acidic Protein (GFAP) within the tumor cells (Fig. 2b, 100× magnification), confirming their astrocytic lineage. The Ki-67 proliferation index (Fig. 2c, 200× magnification) was remarkably low, with only rare tumor cell nuclei showing positive staining, indicating a low proliferative rate at the time of this initial biopsy.

Parents were informed of the brain tumor diagnosis and initially declined surgical resection, consenting only to a simple brain biopsy via burr hole after further discussion. Histopathological examination of the biopsy sample was initially interpreted as a low-grade pediatric glioma, characterized by GFAP and OLIG2 expression with a Ki-67 index <5 % (Fig. 2). The patient was then scheduled for adjuvant therapy and referred to medical oncology.

**Fig. 3:**
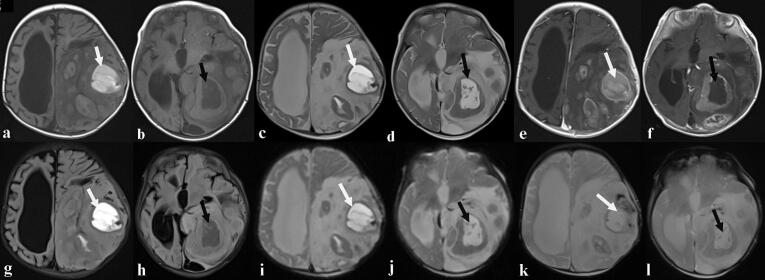
Axial MRI showing the large intra-axial tumor occupying almost the entire left hemisphere measuring approximately 11 × 9 cm in the axial plane and 11 cm in the sagittal plane. It had a heterogeneous signal on both T1 (**a**, **b**) and T2 weighted sequences (**c**, **d**), creating largely necrotic masses (**b**, **d**, **f**, **h**, **l**, black arrows) with hemorrhagic component (**a**, **c**, **e**, **g**, **i**, **k**, white arrows) and heterogeneous enhancement after gadolinium chelate injection (**e**, **f**). It is surrounded by perilesional edema responsible for a discrete midline shift to the right (**g**, **h**). The diffusion weighted sequence (**i**, **j**) showed a heterogeneous signal with hemorrhagic changes on the gradient echo sequence (**k**).

**Fig. 4:**
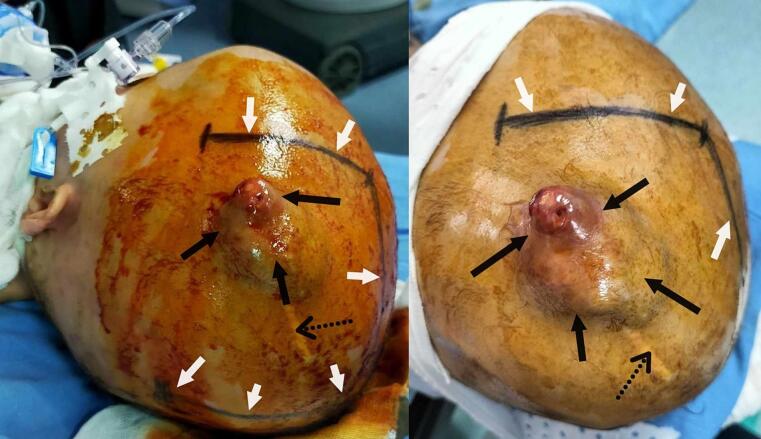
Preoperative photograph showing patient's head fixed in a classic horseshoe headrest and the c-shaped skin incision (white arrows) centered on the exteriorization point of the tumor through the calvarial defect and the skin (black arrows) at a distance from the operating scar of her old biopsy (black dotted arrows).

Unfortunately, the patient was lost to follow-up for over six months. She was readmitted with a recurrence of her previous symptoms and a notable protrusion of the tumor through the skin, distinct from the original biopsy site. Clinical examination again revealed signs of raised ICP, with a friable, easily bleeding mass exteriorizing through a calvarial defect separate from the initial trephine hole (Fig. 4). A repeat MRI (Fig. 3) demonstrated a large (approximately 11 × 9 × 11 cm), intra-axial tumor occupying almost the entire left hemisphere. The lesion displayed heterogeneous signal intensity on both T1- and T2-weighted sequences, with large necrotic and hemorrhagic components and heterogeneous post‑gadolinium enhancement. Significant perilesional edema caused a discrete midline shift to the right and left uncal herniation with mass effect on the mesencephalon. Additionally, cortical signal abnormalities with diffusion restriction (hypersignal on DWI with a low ADC) were observed in the left parietal, right frontal, occipital, hippocampal, and ipsilateral mesial temporal regions.

**Fig. 5:**
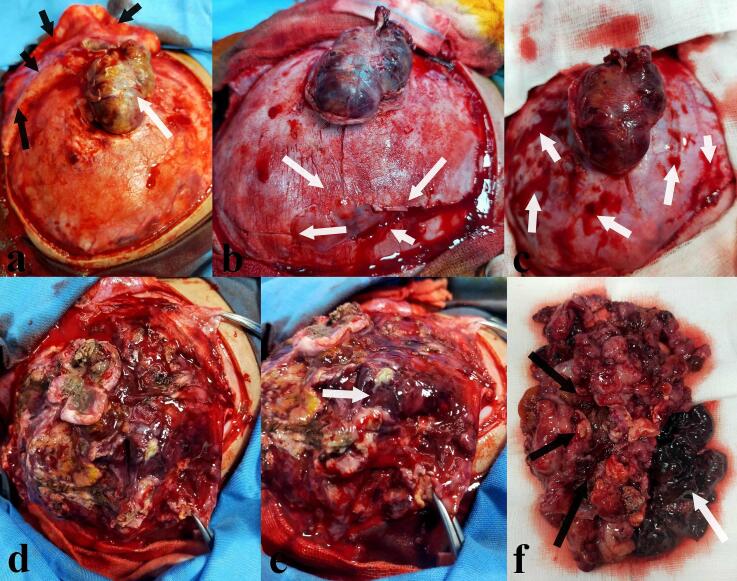
Intraoperative photograph showing the exocranial tumor (**a**, white arrow) after gentle detachment of various subcutaneous tissues (**a**, black arrow). The bone flap all around appeared very thin and fragile and even lysed in places (**b**, white arrows) and was bleeding with the dura which looks quite thin and transparent visualizing the underlying cerebral cortex (**c,** white arrows). The tumor appeared grayish, yellowish, brownish and also reddish in places (**d, e**). There was a large hematoma in the center (**e**, white arrow). The sample sent for anatomopathological study was made of tumor (**f**, black arrows) and hematoma (**f**, white arrow).

**Fig. 6:**
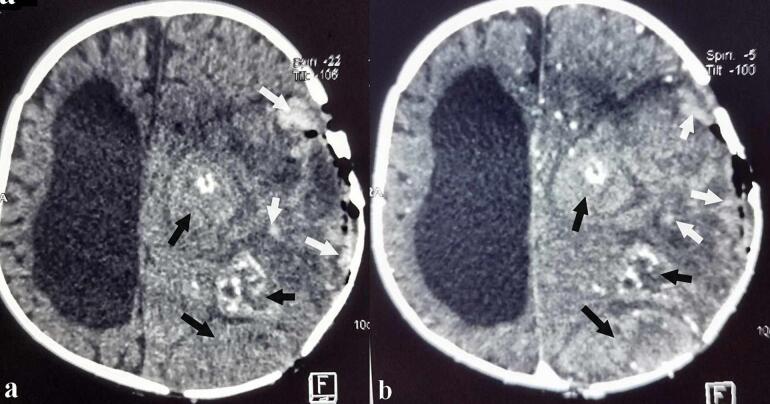
Axial CT scan in parenchymal window performed 24 h postoperatively before (**a**) and after enhancement (**b**) showing the tumor residue (black arrows) with some hemorrhagic foci (white arrows). Note the presence of fine calcifications not clearly visible on preoperative MRI scans and the calvarial defect opposite the pneumocepalus.

After re-discussion, the parents finally provided written informed consent for surgical resection. In the operating room, with the patient supine and head secured, a C-shaped skin incision centered on the tumor exteriorization was made for maximal exposure (Fig. 4). Careful dissection of subcutaneous tissues (superficial fascia, hypodermis, subcutis, galea) was performed to minimize bleeding from the exocranial component. The bone flap surrounding and opposite the tumor was remarkably thin, fragile, and lysed, fracturing during craniotomy. A star-shaped dural opening was made, centered on the lesion. The dura mater appeared thickened, tumor-infiltrated, and intimately adherent. Surgical excision was challenging due to significant bleeding from minimal manipulation. The tumor presented with a variegated appearance (grayish, yellowish, brownish, reddish) (Fig. 5). A large central hematoma was gently evacuated to facilitate brain relaxation. Further dissection revealed a 5–10 ml collection of yellowish, viscous fluid consistent with an abscess, which was sampled for microbiological analysis. This abscess correlated with the necrotic areas seen on MRI. The surgical site was copiously irrigated with hydrogen peroxide to address potential anaerobic bacteria and aid in hemostasis, which was difficult and led to hypovolemia and severe hypotension, requiring massive blood transfusion. Due to these complexities, surgical excision was deemed incomplete. The dura was meticulously curetted and closed primarily without duraplasty. The fractured bone flap was gently replaced and covered by the galea. All tissue samples were fixed in formaldehyde for histopathological examination. The postoperative course was uneventful. A postoperative CT scan (Fig. 6) performed 24 h later showed residual tumor with hemorrhagic foci and fine calcifications.

**Fig. 7:**
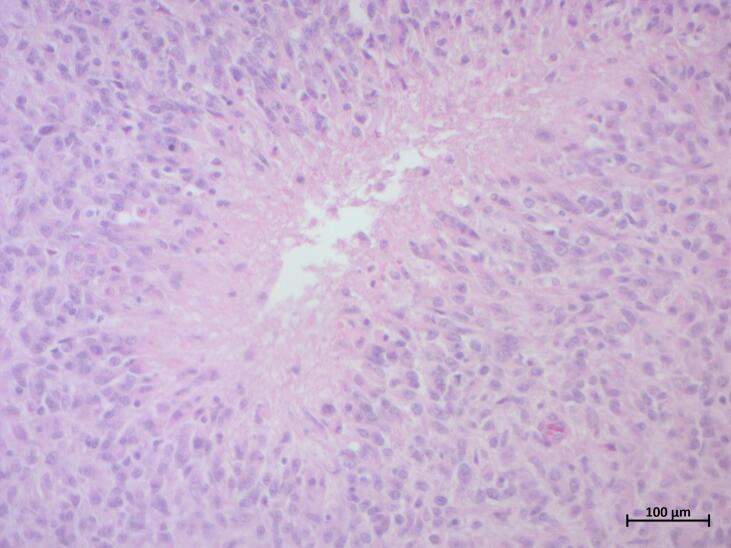
Photomicrograph of the final surgical specimen (Hematoxylin and Eosin staining, 200× original magnification) (Fig. 7a) revealed a high-density proliferation of pleomorphic glial cells with increased cellularity compared to the initial biopsy. Distinct foci of tumor necrosis were prominent, characterized by acellular areas surrounded by pseudopalisading of tumor cells, a hallmark feature of high-grade gliomas. Increased mitotic activity and nuclear pleomorphism were also evident (Fig. 7b, 200× magnification, inset showing mitotic figure), contrasting with the low proliferative index observed in the initial biopsy.

Final histopathological examination of the surgical specimen (Fig. 7) revealed a highly cellular proliferation of ovoid tumor cells with indistinct cytoplasmic borders and occasional gemistocytic features. Cytonuclear atypia was mild, but mitotic activity was high (15 mitoses/2 mm$^2$). Numerous foci of necrosis with pseudopalisading were observed, without prominent vascular proliferation. The tumor was richly vascularized, with extensive hemorrhagic changes, calcifications, and a foreign body giant cell reaction. These features were consistent with a high-grade glioma.

Postoperatively, the infant received antibiotic therapy and was transferred to the pediatric department on postoperative day 5. The final microbiological analysis of the abscess fluid showed no bacterial growth (culture-negative abscess). She was subsequently referred to the medical oncology department for chemotherapy.

## 3 Discussion

Since the 1979 classification, age has become crucial in glioma classification, necessitating the distinction between “pediatric type” and “adult type” gliomas. In children, low-grade gliomas predominantly include pilocytic astrocytomas, gangliogliomas, and dysembryoplastic neuroepithelial tumors. In contrast, “pediatric type” high-grade gliomas (HGGs) are often characterized by histone H3 K27 alterations, while “adult” HGGs typically harbor IDH 1 or 2 mutations. Although these molecular profiles are key differentiators, some overlap between age groups is possible [11].

The incidence and histological distribution of pediatric CNS tumors vary [12]. The Pediatric Oncology Group (POG) identified HGGs as the fourth most common malignant brain tumor in very young children [13], with two-thirds occurring in infants under 6 months, consistent with our patient's age at presentation [13]. Similarly, the Children's Cancer Group (CCG) reported a clustering of HGGs in early life, with a significant proportion diagnosed in the first year [14]. In this very young population, HGGs commonly arise in the cerebral hemispheres [15], as observed in our case, aligning with POG and CCG studies [13,14].

At the histopathological level, pediatric HGGs are frequently associated with histone H3 mutations, which are rare in adults. The 2021 WHO classification redefined glioblastoma to apply exclusively to IDH-wild-type adult diffuse astrocytic glioma, excluding pediatric gliomas. Specific entities like H3 G34-mutated diffuse hemispheric gliomas primarily affect adolescents and young adults. IDH-wildtype and H3-wildtype pediatric diffuse HGGs are grade 4 tumors, diagnosed based on histology and molecular markers (PDGRA, MYCN, EGFR, methylome) [16,17]. Infantile hemispheric gliomas, a newly recognized high-grade entity in newborns and infants like our patient, exhibit distinct molecular profiles involving gene fusions of ALK, ROS1, NTRK, or MET [16,17]. While molecular characterization offers superior prognostic value compared to histology alone [18], this analysis was unfortunately not feasible in our resource-limited setting. The eventual diagnosis of HGG in our patient was based on histopathological findings after the second surgery.

The clinical presentation of infantile glial lesions is influenced by tumor location and patient age [7]. However, the elasticity of the infant cranium can delay symptom onset, allowing significant tumor growth before manifestation [19]. Common initial symptoms include increased intracranial pressure, as seen in our patient, potentially accompanied by focal neurological deficits [8]. In very young children, symptoms can be nonspecific (macrocephaly, vomiting, irritability, lethargy, growth failure) [7,20], potentially delaying diagnosis. In our 5-month-old patient, the initial presentation, while including raised intracranial pressure and macrocrania, was atypical due to the subsequent development of a giant, osteolytic tumor with transcalvarial herniation – a combination, to our knowledge, not previously reported in the pediatric literature.

Radiologically, HGGs typically appear as irregular, heterogeneous lesions with poorly defined borders and nodular enhancement, often surrounded by significant edema [21]. On MRI, they usually show hypointense signals on T1-weighted images and hyperintense signals on T2-weighted images [21]. A significantly reduced T2 signal is rare due to high cellularity, potentially complicating differentiation from embryonal tumors. While most HGGs enhance intensely after gadolinium, the degree of enhancement doesn't always correlate with histological grade [21]. In our case, the initial MRI showed a large lesion with mixed T1 and heterogeneous T2 signals, heterogeneous enhancement with necrosis, and mild edema. The follow-up MRI revealed extensive necrosis with hemorrhagic components and heterogeneous enhancement, consistent with the subsequent finding of a culture-negative abscess within the tumor. Notably, intratumoral abscess formation in pediatric HGGs is exceedingly rare, with all previously reported cases occurring in adults [9,22]. The absence of bacterial growth in our patient's abscess suggests a sterile process, potentially linked to extensive tumor necrosis and hemorrhage, or possibly influenced by the preceding maternal intra-amniotic infection, although this remains speculative. Cerebrospinal fluid (CSF) dissemination occurs in approximately 3 % of pediatric HGGs [23], with variable incidence in very young children [24]. Spinal MRI was not performed in our patient due to concerns about repeated anesthesia in the immediate postoperative period. Importantly, CSF dissemination does not appear to confer a survival disadvantage [13,25].

Surgical resection, aiming for maximal safe removal, is the cornerstone of HGG treatment [26]. However, achieving complete resection in children with often large tumors [27] is challenging and carries a high risk of intra- and postoperative bleeding [28]. This is particularly true for young infants with limited blood volume, making them vulnerable to hypovolemia and cardiac arrest [28]. Our patient with a giant tumor experienced significant bleeding requiring massive transfusion, necessitating an incomplete resection. This aligns with many series reporting complete resection in only about one-third of pediatric HGG cases [13,14,29,30]. The extent of resection is a crucial prognostic factor, with studies showing improved progression-free survival in patients with more extensive resection [31].

Adjuvant therapies, especially radiotherapy, pose significant challenges in young children due to potential long-term sequelae [32]. Patients under 3 years, like ours, are particularly vulnerable [13,32]. Chemotherapy-based regimens are often used to delay radiotherapy in this age group [13,14,29,30]. The CCG-943 trial demonstrated improved event-free survival with adjuvant radiotherapy combined with chemotherapy in older children and adolescents [33], leading to the exploration of various chemotherapy protocols [13,29,34]. In very young patients, some studies suggest that chemotherapy alone can maintain long-term survival [13,14,29,30]. Our patient received antibiotic therapy postoperatively and was referred for chemotherapy without immediate radiotherapy.

This case report presents the first documented instance of a giant, osteolytic pediatric high-grade glioma with transcalvarial herniation and an intratumoral abscess in a 5-month-old infant. The unique combination of these rare features underscores the atypical and extremely rare nature of this presentation, enriching the existing literature and highlighting the potential for unexpected complexities in pediatric HGGs, even in very young patients.

## 4 Conclusion

In conclusion, we present a unique and previously unreported case of a giant, osteolytic pediatric high-grade glioma complicated by transcalvarial herniation and an intratumoral abscess in a 5-month-old infant. While high-grade gliomas in very young children are already relatively rare and challenging to treat, the occurrence of an intratumoral abscess further compounds the diagnostic complexity, highlighting the crucial role of multimodal brain imaging. The management strategy in such cases relies on the most complete surgical resection feasible, followed by chemotherapy to potentially reduce and delay radiotherapy, given its significant risks in this age group. The extreme rarity of this presentation underscores the need for additional trials and collaborative efforts to further elucidate the molecular, genetic, and epigenetic characteristics of these tumors in very young children. Such research is essential for identifying novel therapeutic targets and ultimately improving the management and prognosis for this vulnerable population.

## Consent

Written informed consent was obtained from the patient's legal guardian for publication of this case report and accompanying images.

## Ethical approval

The study is exempt from ethnical in our institution (University of Sfax).

## Funding

There is no sources of funding for this research.

## Author contribution

Mehdi Borni, Marouen Taallah, Brahim Kammoun, Hatem Daoud: study concept, data interpretation.

Mehdi Borni, Houda Belmabrouk, Mohamed Zaher Boudawara: writing the paper.

Mohamed Zaher Boudawara; study concept.

Mohamed Zaher Boudawara: validation.

## Guarantor

Mehdi Borni.

## Research registration number

Not applicable for this article.

## Conflict of interest statement

The authors declared no potential conflicts of interests with respect to research, authorship and/or publication of the article.
